# New Books

**Published:** 2005-01

**A History of Epidemiologic Methods and Concepts**

Alfredo Morabia, ed.

New York:Springer-Verlag, 2004. 405 pp. ISBN: 3-7643-6818-7, $119

**Agriculture and the Nitrogen Cycle: Assessing the Impacts of Fertilizer Use on Food Production and the Environment**

Arvin R. Mosier, Keith Syers, John R. Freney, eds.

Washington, DC:Island Press, 2004. 374 pp. ISBN: 1-55963-710-2, $40

**Carbon Dioxide Utilization for Global Sustainability**

Sang-Eon Park, Jong-San Chang, Kyu-Wan Lee, eds.

Burlington, MA:Elsevier, 2004. 625 pp. ISBN: 0-444-51600-X, $253

**Cell Surface Receptors: A Short Course on Theory and Methods**

Lee E. Limbird

New York:Springer-Verlag, 2004. 218 pp. ISBN: 0-387-23069-6, $85

**Corporate Environmentalism and Public Policy**

Thomas P. Lyon, John W. Maxwell

Cambridge:Cambridge University Press, 2004. 306 pp. ISBN: 0-521-81947-4, $85

**Electronic Scientific, Technical, and Medical Journal Publishing and Its Implications: Report of a Symposium**

Committee on Electronic Scientific, Technical, and Medical Journal Publishing, The National Academies

Washington, DC:National Academies Press, 2004. 122 pp. ISBN: 0-309-09161-6, $28.25

**G Protein Coupled Receptor-Protein Interactions**

Susan R. George, Brian F. O’Dowd, David R. Sibley, eds.

Hoboken, NJ:John Wiley & Sons, 2004. 320 pp. ISBN: 0-471-23546-6, $149

**Global Carbon Cycle Integrating Humans, Climate, and the Natural World**

Christopher B. Field, Michael R. Raupach, eds.

Washington, DC:Island Press, 2004. 568 pp. ISBN: 1-55963-527-4, $45

**How Cancer Works**

Lauren Sompayrac

Sudbury, MA:Jones and Bartlettt Publishers, 2004. 110 pp. ISBN: 0-7637-1821-1, $26.95

**Make Your Mark in Science: Creativity, Presenting, Publishing, and Patents, A Guide for Young Scientists**

Claus Ascheron, Angela Kickuth

Hoboken, NJ:John Wiley & Sons, 2004. 256 pp. ISBN: 0-471-65733-6, $29.95

**Novel Compounds From Natural Products in the New Millennium: Potential and Challenges**

Benny K-H Tan, Boon-Huat Bay, Yi-Zhun Zhu

River Edge, NJ:World Scientific, 2004. 336 pp. ISBN: 981-256-113-7, $65

**Petit Point: A Candid Portrait on the Aberrations of Science**

Pierre-Gilles de Gennes

River Edge, NJ:World Scientific, 2004. 80 pp. ISBN: 981-256-011-4, $14

**Rachel Carson: A Biography**

Arlene R. Quaratiello

Westport, CT:Greenwood, 2004. 168 pp. ISBN: 0-313-32388-7, $29.95

**Science and Technology in the National Interest: Ensuring the Best Presidential and Federal Advisory Committee Science and Technology Appointments**

Committee on Ensuring the Best Presidential and Federal Advisory Committee Science and Technology Appointments

Washington, DC:National Academies Press, 2004. 224 pp. ISBN: 0-309-09512-3, $50.50

**Setting Priorities for Large Research Facility Projects Supported by the National Science Foundation**

Committee on Setting Priorities for NSF-Sponsored Large Research Facility Projects, National Research Council

Washington, DC:National Academies Press, 2004. 236 pp. ISBN: 0-309-09084-9, $41.50

**The Genetic Code and the Origin of Life**

Lluis Ribas de Pouplana, Godfrey Maurice, eds.

New York:Springer-Verlag, 2004. 270 pp. ISBN: 0-306-47843-9, $145

**Teen Guides to Environmental Science**

John Mongillo, Peter Mongillo

Westport, CT:Greenwood, 2004.

Five Volumes, ISBN: 0-313-32183-3, $249.95

